# An In Silico Classification Model for Putative ABCC2 Substrates

**DOI:** 10.1002/minf.201200049

**Published:** 2012-08-09

**Authors:** Marta Pinto, Michael Trauner, Gerhard F Ecker

**Affiliations:** a University of Vienna, Department of Medicinal Chemistry, Althanstraße 14, A-1090 Vienna, Austria phone/fax: +431-4277-55110/-9551; b Division of Gastroenterology and Hepatology, Department of Internal Medicine III, Medical University of Vienna, A-1090 Vienna, Austria

**Keywords:** ATP-binding cassette transporter, ABCC2/MRP2, Machine learning methods, Substrate prediction

ABCC2 (MRP2; also known as cMOAT or cMRP) belongs to the adenosine triphosphate (ATP)-binding cassette (ABC) transporter superfamily, which represents a large family of transmembrane proteins that use the energy of ATP hydrolysis to transport a wide variety of physiological substrates across biological membranes.^1^ Phylogenetically, ABC transporters are classified into seven subfamilies of 49 transporter genes (ABCA to ABCG).^2^ The major physiological role of these transporters is to protect cells and tissues against xenobiotics. Consequently, they also play a critical role in the disposition of drugs and their metabolites, altering their pharmacokinetics and pharmacological profile.^1^

ABCC2, a member of the multidrug resistance-associated protein (MRP/ABCC) subfamily, is strongly expressed on the apical canalicular membrane of hepatocytes,^3^ where it pumps endogenous metabolites conjugated to sulfate, glucuronate or glutathione (GSH) into the bile.^4^ One of the most prominent endogenous substrates are conjugates of bilirubin which results from heme metabolism during breakdown of hemoglobin. Hereditary and acquired ABCC2 defects resulting in impaired bilirubin excretion into bile give rise to jaundice (yellow discoloration of skin and sclera/eyes) as one of the most prominent clinical signs of liver.^5^ Through facilitation of biliary GSH excretion, ABCC2 is one of the critical determinants maintaining (bile acid-independent) bile flow.^5^ Moreover, many nonconjugated xenobiotics,^6^ including unmodified anionic drugs,^7^ are also efficiently transported by ABCC2.

However, in spite of this broad substrate specificity, the molecular determinants of ABCC2-mediated transport are still unknown, probably due to the lack of a high-resolution crystal structure and the possible existence of several binding sites in this protein.^8^ In this scenario, ligand-based methods provide an alternative way to characterize and identify potential ABCC2 substrates. Here, we report the application of different machine learning methods for the development of models for putative substrate/non-substrate classification.

Currently there is no consistent published data set comprising ABCC2 substrates and non-substrates. This is mainly due to the fact that there is no standardized assay for ABC-transporter substrates. Polarized transport across cell-monolayers, cellular uptake, toxicity ratio in overexpressing vs. wild type cells, as well as ATPase stimulation are just a few methods to assess the substrate properties of compounds. Moreover, different assays might lead to different class labels for the same compound. When searching publicly available databases such as ChEMBL and TP-Search,^9^ the use of both databases led to a data set formed by 44 unique compounds, which were not enough to cover a significant portion of the chemical space. Thus, this set of compounds was kept and used in a further step as external data set to validate the model.

In the present work, we used the data set from Szakács et al. to the develop a ABCC2 substrate classification model.^10^ The authors measured the activity of 1429 compounds against 60 tumor cell lines expressing different levels of ABC transporters. By correlating the mRNA level of each transporter with the cytotoxicity of each compound over all 60 cell lines, it was possible to postulate putative substrates for each ABC transporter. Compounds whose cell toxicity is decreased in cells expressing high levels of an ABC transporter may be considered as substrates. Conversely, compounds for which no negative correlation between cytotoxicity and ABC expression level is observed were considered as non-substrates. This data set of course is a quite noisy and fuzzy one, and compounds showing a negative correlation between cytotoxicity and ABCC2 mRNA expression not necessarily are true substrates of ABCC2. However, this dataset represents the largest one available. In addition, it can easily be expanded to the whole NCI60 screening set comprising more than 30.000 compounds.

The original database was carefully curated leading to a final data set formed by 1204 compounds (see Methods). These compounds were classified as substrates or non-substrates based on their Pearson correlation coefficient (hereafter referred to as PCC) values. Two different PCC vales were tested: −0.25 and −0.30. Compounds having a PCC value lower or equal to this threshold value were considered as putative substrates. Conversely, those having a PCC higher than this threshold were categorized as non-substrates. This procedure yielded a data set formed by 1067 non-substrates and 156 substrates for a PCC value of −0.25 and by 98 substrates and 1106 non-substrates when a PCC value of −0.30 was considered (Table 1).

**Table 1:** Composition of the training and test sets considered in the present work. S: substrates; NS Non-substrates.

PCC		Total	TR	TS
		S	NS	S	NS	S	NS
	MACCS	154	1050	126	838	28	212
0.25	Descriptors	154	1050	123	841	31	209
	Random	154	1050	123	840	31	210
	MACCS	98	1106	85	879	13	227
0.30	Descriptors	98	1106	80	884	18	222
	Random	98	1106	78	962	20	222

Taking into account the lack of information about the stereochemistry for most of the compounds, we decided to use only 2D descriptors. These descriptors were calculated using MOE2010 (Molecular Operating Environment; Chemical Computing Group Inc., 2010). A total of 93 different 2D descriptors (see Methods) were computed for all 1204 compounds and subjected to Z-Score normalization. Then, the two resulting data sets (i.e., the data sets obtained for PCC values of −0.25 and −0.30, respectively) were divided into training (TR) and test set (TS) using three different procedures: (1) Diverse Subset module of MOE2010 on basis of the Tanimoto coefficient using MACCS structural keys; (2) MOE2010’s Diverse Subset module using the Tanimoto coefficient and each the set of descriptors previously calculated and, (3) randomly by using KNIME v.2.3.4 (http://knime.org). Consequently, three different sets composed by TR and TS were obtained for each data set, giving a total of 6 different data sets to be used as starting point for developing the models (Figure 1).

For each data set, first a feature selection algorithm was applied in order to reduce the dimensionality of the data by identifying the most relevant features or descriptors. For this purpose, the CfsSubsetEval-Bestfirst algorithm implemented in WEKA was used (http://www.cs.waikato.ac.nz/∼ml/weka).^[11]^ Subsequently, five different WEKA classifiers were tested: Naïve Bayes (NB), *k*-nearest neighbors classifier (IBk), the decision trees J48 and Random Forest (RF) and the Support Vector Machine (SMO).^12^

In order to deal with the imbalance of the data sets (Table 1), a misclassification cost was applied to each classifier using the cost sensitive classifier as implemented in WEKA. This approach increases the cost for misclassifying any minority class (substrate) as the majority class (non-substrate).^12^ Several cost values, arbitrarily assigned, were tested. In addition, the cost sensitive classifier was also applied in combination with Bagging, which samples subsets from the training set, builds multiple base learners and aggregates their predictions to make final predictions.^13^

On the other hand, taking into account the ability of ABCC2 to transport negatively charged compounds, we decided to investigate whether the presence of compounds in the data set possessing a net charge different from zero affected the performance of the model. As the curation protocol neutralizes the compounds, a new database was created by changing the protonation state of strong acids and bases of the initial curated database. Subsequently, the same procedure as described in Figure 1 was applied to the resulting data set.

**Figure 1:**
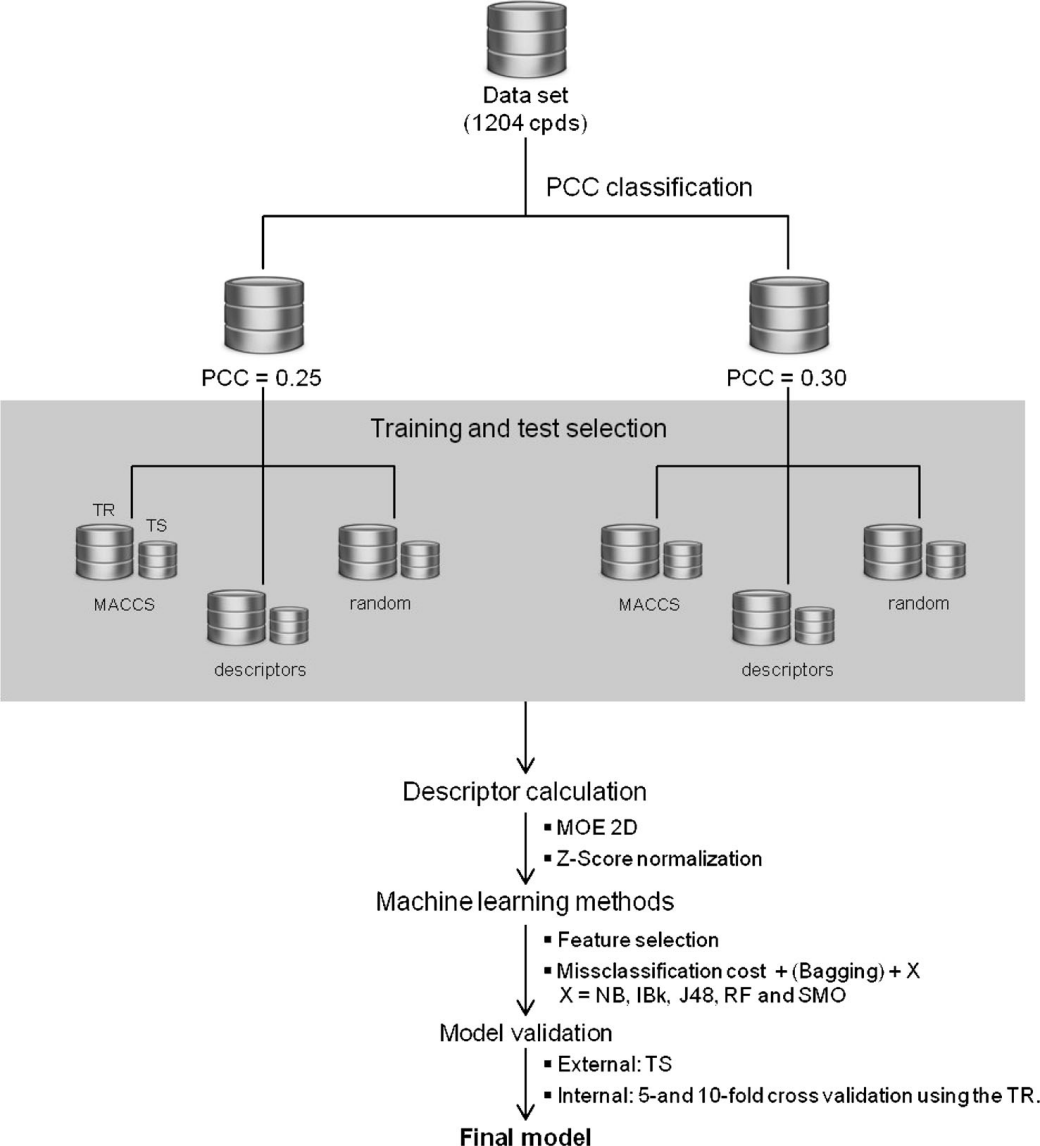
Schematic representation of the procedure used to build models for ABCC2 substrate prediction.

Table 2 shows the best models obtained for the neutral and the charged data sets at PCC values of −0.25 and −0.30, respectively. As can be seen, the addition of charges does not significantly improve the performance of the models, but modifies the type and number of descriptors selected to build the model (Table 3).

**Table 2:** Performance of the best models obtained for the noncharged and charged initial databases at the PCC values considered in the present work. Bottom grey and white have been used to indicate the models obtained at PCC values of 0.25 and 0.30, respectively.

	TR/TS selection	Misclassification cost (FN : FP)	Machine learning algorithm	Specificity	Sensitivity	Precision	G-mean	MCC	Accuracy
Noncharged	Random	65 : 2.5	Bag^[a]^+J48	72.38	70.97	0.28	0.72	0.31	71.67
5-fold (TR)				72.62	67.48	0.27	0.70	0.29	70.05
10-fold (TR)				72.86	65.85	0.26	0.69	0.28	69.36
Charged	Random	150 : 3.5	RF^[b]^	71.90	77.42	0.29	0.75	0.35	74.66
5-fold (TR)				67.26	73.17	0.36	0.70	0.28	70.22
10-fold (TR)				67.26	76.42	0.37	0.72	0.30	71.84
Noncharged	Descriptors	81 : 1.20	Bag+J48	75.23	77.78	0.20	0.76	0.31	76.50
5-fold (TR)				67.76	68.76	0.16	0.68	0.21	68.26
10-fold (TR)				72.62	62.50	0.17	0.67	0.21	67.56
Charged	Descriptors	80 : 1.10	RF	73.87	77.78	0.19	0.76	0.30	75.83
5-fold (TR)				66.52	68.75	0.16	0.68	0.20	67.63
10-fold (TR)				71.27	67.50	0.18	0.69	0.23	69.38

[a] *Bag* has been used to design the *bagging* algorithm. [b] *RF* refers to the Random Forest machine learning method.

**Table 3:** Set of 2D MOE2010 descriptors used to build each one of the models shown in Table 2.

Descriptor	Description	PCC=0.25	PCC=0.30
		Noncharged	Charged	Noncharged	Charged
a_don	Number of hydrogen bond donor atoms.	**√**	**√**		**√**
a_nBr	Number of bromine atoms.	**√**	**√**		
a_nCl	Number of chlorine atoms.	**√**	**√**	**√**	**√**
a_nN	Number of nitrogen atoms.	**√**			
a_nO	Number of oxygen atoms.			**√**	**√**
a_nS	Number of sulfur atoms.	**√**	**√**		
b_count	Number of bonds.	**√**	**√**		
PEOE_VSA+1	Sum of the van der Waals surface area of atoms having a charge in the range [0.05, 0.10).			**√**	
PEOE_VSA+2	Sum of the van der Waals surface area of atoms having a charge in the range [0.10, 0.15).	**√**	**√**		
PEOE_VSA+3	Sum of the van der Waals surface area of atoms having a charge in the range [0.15, 0.20).	**√**	**√**	**√**	
PEOE_VSA+4	Sum of the van der Waals surface area of atoms having a charge in the range [0.20, 0.25).	**√**	**√**	**√**	
PEOE_VSA_FNEG	Fractional negative van der Waals area.	**√**	**√**	**√**	**√**
PEOE_VSA_FPOS	Fractional positive van der Waals area.				**√**
PEOE_VSA_POS	Total positive van der Waals area	**√**		**√**	**√**
PEOE_VSA_PPOS	Total polar positive van der Waals area				**√**
rings	Number of rings.	**√**	**√**	**√**	
SlogP_VSA0	Sum of the van der Waals surface area of atoms which contribution to logP is ≤−0.40.	**√**	**√**	**√**	**√**
SlogP_VSA1	Sum of the van der Waals surface area of atoms which contribution to logP is in (−0.40, −0.20].		**√**		
SMR_VSA1	Sum of the van der Waals surface area of atoms which contribution to MR is in (−0.11, −0.26].		**√**	**√**	**√**
SMR_VSA2	Sum of the van der Waals surface area of atoms which contribution to MR is in (−0.26, −0.35].		**√**		
SMR_VSA4	Sum of the van der Waals surface area of atoms which contribution to MR is in (−0.39, −0.44].	**√**		**√**	**√**
TPSA	Total polar surface area (connection table approximation)	**√**			
vsa_base	Number of basic atoms.				**√**
vsa_don	Sum of van der Waals surface areas of pure hydrogen bond donor atoms.	**√**	**√**	**√**	**√**
vsa_other	Sum of van der Waals surface areas of atoms typed as “others”.		**√**		

The best model for identification of putative MRP2 substrates was obtained by including explicit charges at PCC value of −0.25. This model, built on 16 2D MOE2010 descriptors using the CostSensitive classifier and Random Forest, was capable to predict correctly 77.4 % and 71.9 % of substrates and non-substrates of the test set, respectively (Table 2).

The 16 2D MOE2010 descriptors used to build this model are briefly summarized in Table 3. As can be seen, the number of rings and bonds, and therefore the molecular size or the molecular weight, are important factors for ABCC2 substrates. Also the number of bromine, chlorine and sulfur atoms plays a critical role. This last is in agreement with the ability of ABCC2 to transport compounds having sulfur atoms in their structures, i.e., conjugate to sulfate or glutathione.

Lipophilicity and low polarizability are also important for ABCC2 recognition. However, ABCC2 substrates have some properties that are distinctly different from those reported for the well-characterized ABC family member ABCB1 (also named P-glycoprotein, P-gp or MDR1).^14^ These properties are the fractional negative charge (PEOE_VSA_FNEG) and vsa_don, which represents the sum of the van der Waals surface areas of all H-bond donors in the molecule. The importance of having a fractional negative charge in the molecule is in agreement with previous experimental studies showing the ability of ABCC2 for transporting anionic compounds and with the results obtained by Ryu et al, who identified the positively charged residues Lys^324^, Lys^483^, Arg^1210^ and Arg^1257^ as critical residues for ABCC2-substrate interaction.^15^

Nevertheless, it is important to note that the fractional negative van der Waals surface area (PEOE_VSA_FNEG), the sum of the van der Waals surface area of atoms which contribution to logP is equal or lower than −0.40 (SlogP_VSA0) and the sum of the van der Waals surface areas of all H-bond donors in the molecule (vsa_don), are always selected by the feature selection algorithm (Table 3), irrespective of the PCC value and the deprotonation state of the molecules. This observation points clearly towards the relevance of these properties in ABCC2 substrate binding.

Finally, we proceeded to evaluate the ability of the model to classify new compounds. To this end, a data set formed by 44 compounds (2 non-substrates and 42 substrates) extracted from TP-Search and PubChem, was used.

In order to assess if these new compounds are within the applicability domain of the training set, it was evaluated by use of principal component analysis (PCA) of the 16 descriptors used to build the model. As can be seen in Figure 2, all the compounds of the external data set can be considered inside the applicability domain of the model.

**Figure 2:**
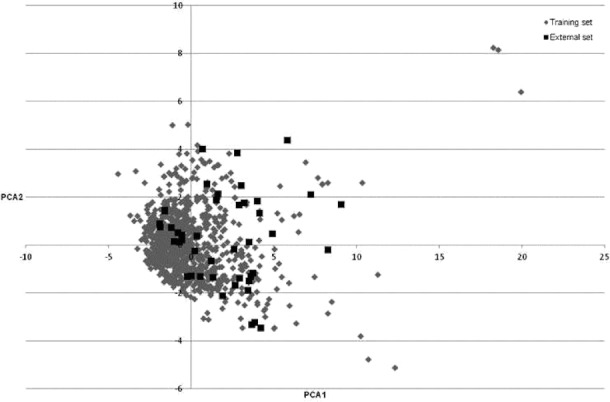
Principal component analysis of the 16 descriptors used to build the model.

The results of the external validation (FN=27, FP=1) clearly showed a low predictive ability of the model; it was only able to correctly predict 26.2 % of the substrates (sensitivity). This drop in predictive power of the model might be attributed to the fact that it was developed for a reversely imbalanced data set. The number of substrates in the training set was lower in comparison with the number of non-substrates. On contrary, the number of substrates in the external data set exceeded largely the number of non-substrates.

Another possible explanation for the results obtained from external validation is that the activity of the compounds used as external set has been measured using different assays and cell lines. Especially in case of substrates one has to be very careful, as different assays (polarized transport, cytotoxicity, ATPase stimulation) might lead to different class labels for the same compound. Thus, strictly spoken our models are able to predict compounds which show a negative correlation of cell toxicity vs. mRNA levels of ABCC2, which not necessarily classifies these compounds as true substrates of ABCC2.

However, the present work represents the first model for classification of a large set of putative ABCC2 substrates and non-substrates. Although the performance is not overwhelming, one should note that a highly imbalanced data set composed of mostly natural product type compounds has been used, which represents an extremely challenging scenario. Analysis of the top scored model shows that the use of charged compounds to build the model does not significantly improve the performance of the model, but clearly modifies its qualitative description. For ABCC2, the most predictive model was obtained by inclusion of explicit charges at PCC values of −0.25. This model shows the importance of the hydrophobicity, the polarizability, the fractional negative charge and H-bond donor properties for compounds showing a negative correlation between their toxicity and the expression rate of ABCC2 over a large panel of tumor cells.

## Computational Methods

*Database preparation.* The database, containing the NSC identifiers for 1429 compounds, was available as supplementary material at the http://www.sciencedirect.com/science/article/pii/S1535610804002065 webpage. Chemical structures of these compounds were retrieved according to their NSC identifier from the NCI database file *NCI_aug00_2D.sdf*, downloaded from the http://cactus.nci.nih.gov/download/nci/ web site. No structure was found for 16 of these compounds. Accordingly, a database formed by 1413 compounds was obtained.

*Database curation.* The database was curated using a multistep procedure based on that proposed by Fourches et al.,^16^ which can be briefly summarized as follows:

*Removal of inorganic compounds* (i.e., compounds not containing carbon atoms in their structure) by calculating the empirical formula of each compound using the Instant Jchem v.5.3 program (http://www.chemaxom.com) and sorting them in ascending order.

*Analysis and removal of mixtures*. Entries formed by more than one compound were decomposed using the Wash module implemented in MOE2010. Each one of the obtained components was stored into separated columns for further analysis. Only mixtures formed by a large organic molecule and a small inorganic one such as sulfates, hydrochlorides, etc., were retained. Mixtures formed by several organic compounds with similar organic weight or by one large organic compound and several smaller ones were deleted due to the impossibility to know which molecule was responsible of biological activity.

*Deletion of organometallics*. Compounds containing metal atoms were identified and deleted using MOE2010.

*Removal of compounds having special atoms*. Additionally, compounds having atoms for which some descriptors cannot be calculated such as tellurium or selenium were identified and discarded using an in-house MOE2010 SVL script.

*Normalization of chemotypes*. Functional groups and tautomeric structures were converted to single standard forms in order to avoid inconsistencies in the calculation of descriptors and also in the elimination of duplicates. This step was performed using the ChemAxon’s Standardizer with the classical settings (clean 2D, aromatize, mesomerize, neutralize, tautomerize and transform options). In addition, the option “clear stereo” was used as first step in order to eliminate any 3D structural information contained in the original .*sdf* file. In this sense, the reader may take into account that there is no definite information about the reliability of the stereochemical data of the compounds in the NCI database.

*Identification and elimination of duplicates*. Nonunique structures were identified using MOE2010. Once identified, PCC values were compared. For compounds having different PCC values, all the related entries were deleted. Otherwise, for identical compounds having similar PCC values, only one entry was kept.

*Removal of compounds containing permanent charges*. Compounds having permanent or non neutralizable charges were identified by calculating the total charge of the molecule using the FCharge descriptor implemented in MOE2010. Compounds with total charge different from zero were discarded.

After this procedure, a data set formed by 1204 compounds was obtained. This data set is available on our website http://pharminfo.univie.ac.at/links/ and on www.chemspider.org.

*Addition of charges.* In order to investigate the effect of including charged compounds on the performance of the model, strong acids and bases of the curated database were deprotonated or protonated, respectively using the *Wash* module of MOE2010.

*Compounds classification.* Members of the dataset were classified as substrate or non-substrates based upon their Pearson correlation coefficient. Two different threshold PCC values were used for classification: −0.25 and −0.30. Compounds with a PCC lower or equal to this threshold value were considered as substrates. Conversely, compounds having a PCC value higher than this value were considered as non-substrates.

*Descriptors calculation.* A set formed by 93 2D descriptors accounting for physical properties (excepting Fcharge, mutagenic, reactive, rsynth and SlogP), subdivided surface areas, atom and bound counts (excepting a_ICM, a_IC, chiral, lip_druglike, lip_violation, Oprea-related, VadjMa and VadjEq), pharmacophore features and partial charges (excepting Q_ and PEOE_PC+, Q_ and PEOE_PC-, Q_ and PEOE_RPC+, Q_ and PEOE_PRC-) was calculated with MOE2010.

*Training and test set selection.* Compounds, sorted in ascending order according to their PCC values, were split into training and test set using three different procedures:

a. A training set, containing 964 compounds (i.e., 80 % of the data set), was generated using the MACCS structural keys and the Tanimoto coefficient similarity metric as implemented in the Diverse Subset feature of MOE2010. The remaining compounds (240 compounds) were selected as test set.

b. A second training and test sets were selected using the Diverse Subset algorithm of MOE2010 based on the Tanimoto coefficient and set of descriptors previously calculated. The composition of each training and test set is shown in Table 1.

c. The data set was divided into substrates and non-substrates. Then, 80 % of each category (i.e., 124 substrates and 853 non-substrates) was randomly selected for use as training set using KNIME v. 2.4.0 (http://www.knime.org/). The remaining compounds were considered as test set. In all the cases, the test set was used to evaluate the performance of the model built on the training set.

*Machine learning.* The selections of the best-performing algorithm and an optimal set of properties for the selected algorithm were performed using WEKA. The CfsSubsetEval-BestFirst method, used as a pre-processing step to machine learning, was applied on the training set in order to select relevant features for the model. Five different machine learning methods, in combination with the Weka’s CostSensitive classifier, were used: Naïve Bayes (NB), *k*-nearest neighbors classifier (Ibk), the Weka’s implementation of a C4.5 decision tree learner (J48), Random Forest (RF) and the Weka’s implementation of the Support Vector Machine (SMO). In all cases, the CostSensitive classifier was also used in combination with Bagging.

*Performance measurement.* The performance of each algorithm was measured using 5- and 10-fold cross-validation analysis. In addition, five performance measures were used: true negative rate (TN) rate (for specificity), true positive (TP) rate (for sensitivity), G-Mean, Matthews Correlation Coefficient (MCC), F-measure and predictive accuracy, as defined below:

Specificity=TN/(TN+FP)

Sensitivity=Recall=TP/(TP+FN)

Precision=TP/(TP+FP)

G-Mean=(Sensitivity×Specificity)^1/2^

MCC=[(TP×TN)−(FP×FN)]/[(TP+FP)(TP+FN)(TN+FP)(TN+FN)]^1/2^

Accuracy=(TN+TP)/(TN+TP+FN+FP)

where we took the minority class (substrates) as positive class.

*External validation data set.* ABCC2 substrates and non-substrates used for external validation were retrieved from TP-Search and ChEMBL. 42 and 10 unique compounds names were obtained from TP-Search and ChEMBL, respectively. No structure was found for 5 compounds of TP-Search, leading a total of 37 chemical structures obtained from this database. Thus, three of the compounds found in ChEMBL were also included in TP-Search. Consequently, a data set formed by 44 compounds (37 from TP-Search and 7 from ChEMBL), of which only two were non-substrates, was used. Chemical structures of these compounds were obtained from Pubchem Compound.^17^ Curation and addition of net charges were performed using the procedure previously described.

*Applicability domain.* The applicability domain of the model was assessed using a principal component analysis. PCA was performed using MOE2010 and the set of descriptors used to build the model.
